# X-ray dichroism in polyimide caused by non-resonant scattering

**DOI:** 10.1107/S1600577520015568

**Published:** 2021-01-01

**Authors:** K. S. Schulze, R. Loetzsch, R. Rüffer, I. Uschmann, R. Röhlsberger, G. G. Paulus

**Affiliations:** a Helmholtz-Institut Jena, Fröbelstieg 3, 07743 Jena, Germany; b Institut für Optik und Quantenelektronik, Friedrich-Schiller-Universität Jena, Max-Wien-Platz 1, 07743 Jena, Germany; c GSI Helmholtzzentrum für Schwerionenforschung, Planckstrasse 1, 64291 Darmstadt, Germany; d ESRF – The European Synchrotron, CS 40220, 38043 Grenoble Cedex 9, France; e Deutsches Elektronen Synchrotron DESY, Notkestrasse 85, 22607 Hamburg, Germany

**Keywords:** polyimide, polarization, X-ray polarimetry, wide-angle scattering, X-ray dichroism

## Abstract

Dichroism is one of the most important optical effects in both the visible and the X-ray range. Besides absorption, scattering can also contribute to dichroism. This paper demonstrates that, based on the example of polyimide, materials can show tiny dichroism even far from electronic resonances due to scattering. Although the effect is small, it can lead to a measurable polarization change and might have influence on highly sensitive polarimetric experiments.

## Abstract 2

Aligned molecules, for example in polyimide foils, lead to small dichroism even far from resonances, which can be revealed by high-precision X-ray polarimetry.

## 1. Introduction   

Dichroism, which is the polarization-dependent absorption of light, is widely used for polarizers in the visible range with applications in physics, chemistry and biology (Nordén & Roger, 1997 ▸). In the X-ray range, many remarkable methods are based on dichroism and provide important insights into matter: X-ray magnetic circular (van der Laan *et al.*, 1986 ▸; Schütz *et al.*, 1987 ▸; Stöhr, 1999 ▸; Funk *et al.*, 2005 ▸; van der Laan & Figueroa, 2014 ▸) and linear dichroism (van der Laan *et al.*, 1986 ▸; Carra *et al.*, 1993 ▸), X-ray natural dichroism (Ade & Hsiao, 1993 ▸; Mainka *et al.*, 1995 ▸; Alagna *et al.*, 1998 ▸; Peacock & Stewart, 2001 ▸; Ney *et al.*, 2014 ▸), and dichroism in the extended X-ray absorption fine structure (Dittmer & Dau, 1998 ▸). The underlying effects can be attributed to the change in the absorption coefficient close to electronic resonances and, thus, provide information about the electronic and magnetic structure of a specific atom.

Far from resonances, optical anisotropies are known to arise in crystals in the vicinity of diffraction peaks. The resulting birefringence and dichroism found application in X-ray phase plates (Skalicky & Malgrange, 1972 ▸) and polarizers (Hasegawa *et al.*, 1999 ▸), respectively. In polymers, dichroism has been observed close to resonances (Collins *et al.*, 2001 ▸), but has rarely been investigated far from it, although there was an attempt to measure X-ray dichroism in Polaroid polarizer sheets (Hart, 1978 ▸). Recent progress in precision polarization analysis has brought non-resonant dichroism into focus. On the one hand, current high-precision X-ray polarimeters are highly sensitive to tiny changes in polarization (Marx *et al.*, 2013 ▸) and, thus, enable the detection of small dichroic effects. On the other hand, dichroism can also be a disrupting factor when studying other polarization changes. In particular, dichroism of optical elements in the beam path such as vacuum windows can influence the measurement and limits its sensitivity.

Like absorption, dichroism is not only based on the photo effect. According to the optical theorem, scattering reduces the transmission through a sample as well and must not be neglected, especially in the X-ray range. A clear example is the aforementioned Bragg diffraction at a crystal with a Bragg angle very close to 45°. Since only the polarization component perpendicular to the plane of diffraction is diffracted, the transmitted beam can only have the remaining polarization direction (Hasegawa *et al.*, 1999 ▸). Polarization changes may also arise in wide-angle X-ray scattering (WAXS). WAXS experiments are usually performed at synchrotron radiation sources, which provide linear polarization. The influence of polarization on the scattering pattern received in these experiments is well known. Thus, the amount of scattered radiation and consequently the absorption depends on the one hand on the structure of the sample and on the other hand on the polarization of the incident beam. Thus, depending on the structure, materials can show linear dichroism from scattering alone.

In this letter, we show that polarization-dependent differences in the scattered intensity far from electronic resonances can lead to small dichroism and, hence, to changes in the polarization of the transmitted beam. This effect is demonstrated using polyimide foil as the sample, a material commonly used as vacuum windows or a substrate in the X-ray range.

## 2. Description of scattering-induced dichroism   

**Figure 1:**
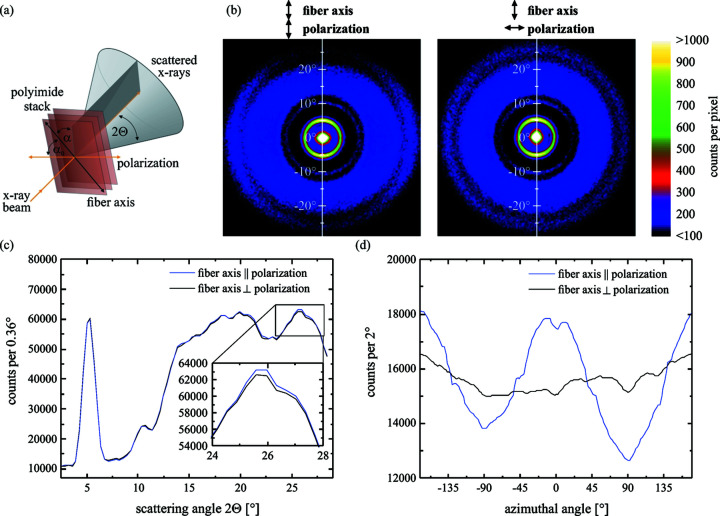
(*a*) Definition of angles used for the analytic description of scattering-induced dichroism. (*b*) WAXS of polyimide Kapton using copper *K*α radiation shows a dependence on the direction of the polarization to the fiber axis. This difference leads to a tiny dichroism for the transmitted beam. The scale is chosen in such a way that tiny differences become visible. The central spot contains about 10^8^ photons. (*c*) Radial dependence of the scattering patterns (azimuthal integral) shows a small difference in scattering at large scattering angles. (*d*) Azimuthal variation of the scattering for both orientations of polarization becomes more clear after radial integration from 2Θ = 8° to 28°. The graphs were smoothed using low-pass filtering.

In order to describe dichroism induced by scattering analytically, one has to consider the dependencies of the scattered intensity. In the kinematical approximation, the intensity can be written as a product of different contributions [see for example the work by Als-Nielsen & McMorrow (2001 ▸)]: 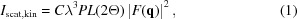
where **q** is the scattering vector, λ is the wavelength and 2Θ is the scattering angle. One contribution is the structure factor *F*(**q**), which describes the influence of the molecular structure of the sample. The geometrical dependence is accounted for by the Lorentz factor *L* and additional dependencies by the parameter *C*. Important for dichroism is the influence of polarization, which is described by the polarization factor *P*. For ideal linear polarization, its behavior is described by [compare with the work by Kahn *et al.* (1982 ▸)] 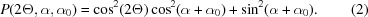
Here, α_0_ is the angle between the polarization of the X-ray beam and a symmetry axis of the sample and α is the angle between this axis and the scattering plane. These angles are explained schematically in Fig. 1 ▸(*a*). Consequently, the sum (α + α_0_) is the angle between the polarization vector and scattering plane. For (α + α_0_) = 0 when the scattering plane is parallel to the polarization vector, the scattered intensity is decreased by a factor of cos^2^(2Θ). If the scattering plane is perpendicular to the polarization vector, this dependency will not exist. The structure factor can be expressed by the scattering angle 2Θ and the angle α: *F*(2Θ, α). The integration of the scattered intensity over all scattering vectors, or rather over all scattering angles and orientations of the scattering planes, gives 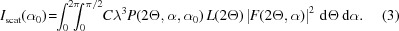
Aside from photoelectric absorption, the incident intensity *I*_0_ decreases by *I*_scat_ during transmission through the sample. Since *I*_scat_ is a function of α_0_ and thus depends on the orientation of the incident polarization, the transmitted intensity is also dependent on it. Therefore, the difference of the scattered intensity, 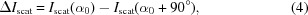
when the incident polarization is rotated by 90°, describes the dichroism of the sample. As expected, Δ*I*_scat_ vanishes for isotropic samples where d*F*/dα = 0, and for samples where the structure factor *F*(2Θ, α) is a function of sin(2α) or its harmonics. In nearly perfectly arranged systems like crystals, dichroism is very strong in the vicinity of diffraction peaks. In non-crystalline materials, the constituents are aligned statistically, but may have a preferred orientation, which gives rise to azimuthal varying scattering.

Polyimides are an example of a plastic showing this behavior, depending on the process of production. In fact, these foils show fiber diffraction (Conte *et al.*, 1976 ▸) with a unique symmetry axis (fiber axis). In the visible range, this symmetry leads to optical anisotropy (Nakagawa, 1990 ▸). The X-ray dichroism of these materials is much weaker compared with crystals, but exists for all orientations of the sample. Close to resonances, this dichroism is superimposed by electronic dichroism. Far from that, it can only be detected by extremely sensitive polarization-resolving devices. Moreover, since polyimides are used as windows for X-rays (*e.g.* vacuum windows, sample holders), these foils can destroy the polarization state and distort polarization-analyzing measurements. For this reason, we first investigated the polarization-dependent scattering of a frequently used polyimide and, second, its influence on the linear polarization of X-rays with the help of a high-purity X-ray polarimeter (Marx *et al.*, 2013 ▸).

## 3. Polarization-dependent WAXS   

Polarization-dependent WAXS was performed with copper *K*α radiation from a microfocus rotating anode X-ray source by Rigaku containing a parallel beam multilayer optic. The beam was polarized by a 1.3 mm-thick silicon crystal used as a Borrmann polarizer (Schulze *et al.*, 2014 ▸). A Pilatus 100K from Dectris was used as a 2D detector for single photon counting and allowed the detection of the scattered photons together with the primary beam because of its high dynamic range of seven orders of magnitude. For the sample, we used a stack of 30 Kapton foils from DuPont, a common polyimide, each 50 µm-thick. The pieces were cut from the same foil, stacked with equal azimuthal orientation and fixed in an aluminium frame afterwards. Since the resulting thickness of 1.5 mm is close to the absorption length of 1.2 mm, maximum scattering intensity was expected. The direction of the fiber axis was determined by a WAXS pattern without a polarizer and by comparison with the data from the work by Conte *et al.* (1976 ▸). Using this knowledge, the fiber axis was aligned with an accuracy of a few degrees to the polarization after the installation of the polarizer crystal.

**Figure 2:**
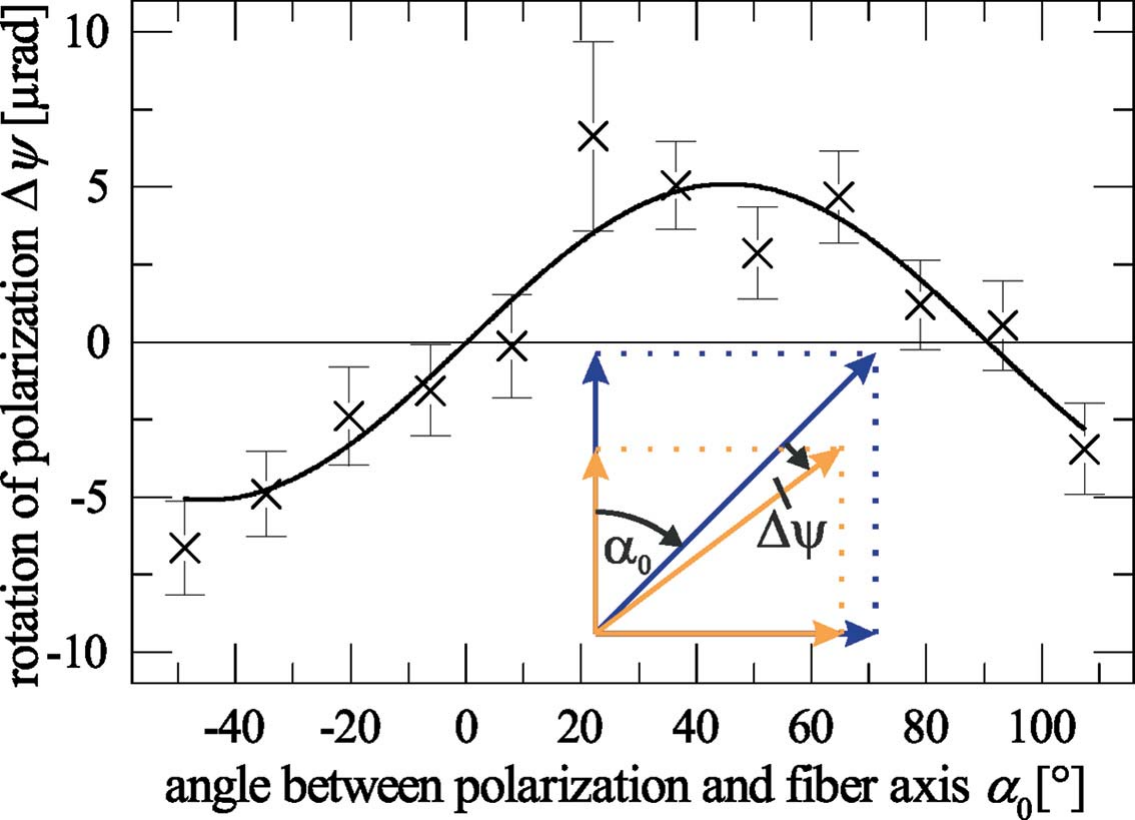
Rotation of the polarization plane for different azimuthal orientations of a stack of Kapton foils caused by dichroism at 14.4125 keV. The relationship between dichroism and rotation of the polarization plane is sketched in the inset. The blue arrows indicate the polarization and its projection parallel and perpendicular to the fiber axis of the incoming beam, and the orange arrows describe the polarization and its projections after passing a dichroic sample.

Fig. 1 ▸(*b*) shows two WAXS patterns with the incident polarization parallel and perpendicular to the fiber axis of the sample. The differences are small but clearly visible between the scattering angles 2Θ = 15° and 30°. The radial integration of the scattering patterns as shown in Fig. 1 ▸(*d*) make this behavior even more clear. The pattern for the perpendicular orientation seems to be independent of the azimuth, whereas the pattern of the parallel orientation shows strong azimuthal behavior. The reason is that the structure factor is approximately a function of sin(α). Its superposition with the polarization factor either enhances or suppresses the sinusoidal behavior of the scattered intensity. As a result, more photons are scattered at large scattering angles when the fiber axis is parallel to the polarization. The radial dependence of the scattered photons displayed in Fig. 1 ▸(*c*) shows the small difference between the two settings. Overall, this leads to a polarization-dependent scattered intensity Δ*I*_scat_ normalized to the transmitted intensity *I*_0_ of 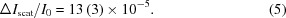
To measure such a small difference of the transmitted intensity for two different polarization states, about 10^10^ photons are required. In this case, the incident beam on the sample has to be stable in the order of the effect, or its flux has to be measured with the corresponding accuracy. However, one can use the fact that dichroism leads to a rotation of the polarization plane as sketched in the inset of Fig. 2 ▸. This rotation is independent of the actual flux of the incident beam and thus demands a less harsh beam stability. From simple trigonometric considerations, one finds that the rotation of the polarization plane Δψ is 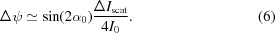
According to the WAXS data, the maximum rotation should be Δψ = 32.5 (8) µrad for copper *K*α when the polarization is 45° to the fiber axis. Such small polarization changes can be measured with the current high-purity polarimeters (Marx *et al.*, 2013 ▸).

## 4. Dichroism of polyimide   

The polarimetry experiment was performed at the nuclear resonance beamline ID18 of the ESRF (Rüffer & Chumakov, 1996 ▸). A photon energy of 14.4125 keV was chosen, the energy of the Mössbauer transition of the iron isotope ^57^Fe, which is an important application of these polarimeters (Toellner *et al.*, 1995 ▸; Siddons *et al.*, 1995 ▸; Röhlsberger *et al.*, 1997 ▸; Heeg *et al.*, 2013 ▸, 2015 ▸; Haber *et al.*, 2016 ▸). A polarization analyzer was used to measure the change of polarization for different orientations of the fiber axis to the polarization of the incident beam.

Owing to the high polarization purity of 10^−8^ provided in this experiment, small changes in the microradian range could be measured, as shown in Fig. 2 ▸. The sinusoidal behavior of the rotation of the polarization plane in terms of dependence on α_0_ as expected by equation (6) is clearly visible. The maximum rotation of 5.3 (6) µrad corresponds to a relative intensity difference of 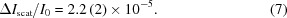
The difference in the value of equation (5) results from the higher photon energy in the polarimetric experiment, which decreases the scattered intensity and the scattering angles. The latter changes the dichroism by just a few percent. However, the scattered intensity scales with the third power of the wavelength according to equation (1). In our case this leads to a reduction of the scattered intensity by a factor of 5.7. In the same way, dichroism is reduced, which is in agreement with the results. The transmittance of the 30 µm × 50 µm-thick polyimide stack was measured to be 78 (1)%. Together with the difference in transmission in equation (7), the relative difference of the absorption coefficient μ of Kapton at 14.4125 keV yields 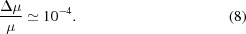



## 5. Conclusions   

Polarization-dependent scattering at samples with aligned molecules leads to a change of the polarization plane after the sample. A single sheet of 50 µm-thick Kapton would rotate the polarization plane by about 0.2 µrad at 14.4125 keV. In most experiments, such a tiny change will not have any influence on the results. If multiple foils are stacked randomly with respect to their fiber axis, the effect is suppressed even further. However, high-precision measurements of the polarization are considered for the investigation of different fundamental physical questions such as the detection of vacuum birefringence (Heinzl *et al.*, 2006 ▸; Karbstein *et al.*, 2015 ▸) or the measurement of magnetic fields in solid density plasma (Huang *et al.*, 2017 ▸). For such experiments, the influence of every element in the beam path has to be considered. Materials that show a stronger alignment of molecules are expected to show a much higher dichroism and, hence, have to be avoided in precision polarimetry. The proof of relationship between molecular order and polarization change is therefore an important step for the development of future high-sensitivity polarimetric setups.
